# Local biomass burning is a dominant cause of the observed precipitation reduction in southern Africa

**DOI:** 10.1038/ncomms11236

**Published:** 2016-04-12

**Authors:** Øivind Hodnebrog, Gunnar Myhre, Piers M. Forster, Jana Sillmann, Bjørn H. Samset

**Affiliations:** 1 Center for International Climate and Environmental Research-Oslo (CICERO), P.O. Box 1129 Blindern, N-0318 Oslo, Norway; 2 School of Earth and Environment, University of Leeds, Leeds LS2 9JT, UK

## Abstract

Observations indicate a precipitation decline over large parts of southern Africa since the 1950s. Concurrently, atmospheric concentrations of greenhouse gases and aerosols have increased due to anthropogenic activities. Here we show that local black carbon and organic carbon aerosol emissions from biomass burning activities are a main cause of the observed decline in southern African dry season precipitation over the last century. Near the main biomass burning regions, global and regional modelling indicates precipitation decreases of 20–30%, with large spatial variability. Increasing global CO_2_ concentrations further contribute to precipitation reductions, somewhat less in magnitude but covering a larger area. Whereas precipitation changes from increased CO_2_ are driven by large-scale circulation changes, the increase in biomass burning aerosols causes local drying of the atmosphere. This study illustrates that reducing local biomass burning aerosol emissions may be a useful way to mitigate reduced rainfall in the region.

## Abstract (web summary)

Black carbon aerosols in the atmosphere absorb solar radiation and affect the hydrological cycle. Here, the authors show that local aerosol emissions from biomass burning activities are a main cause of observed decline in southern African dry season precipitation over the last century.

## Graphical abstract

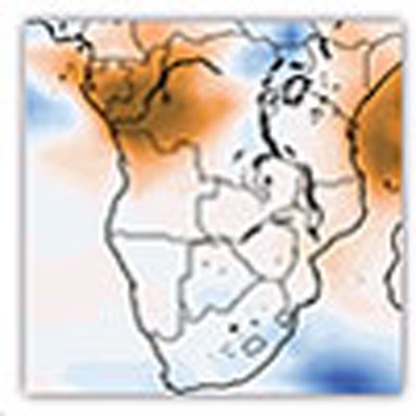

Black carbon (BC) aerosols, in the atmosphere, absorb solar radiation and may affect the hydrological cycle through changes in the atmospheric and surface energy budgets^1^,^2^,^3^,^4^,^5^. In contrast to most greenhouse gases, BC is removed from the atmosphere only after a few days^6^,^7^. Absorbing aerosols such as biomass burning BC modify cloud distribution, mainly by suppressing convective activity, albeit with some debate existing in the literature^8^,^9^,^10^,^11^,^12^. On a global scale, the altitude of added absorbing aerosol concentration is shown to influence the magnitude and sign of global average precipitation changes, with a net decrease in precipitation expected unless BC is added close to the surface^3^,^13^,^14^,^15^,^16^,^17^. Organic carbon (OC) aerosols are emitted together with BC and have approximately the same lifetime, but OC scatters sunlight to a much larger extent than BC, and therefore cools the atmosphere-surface system^6^. Biomass burning is a major source of BC and OC aerosols and normally takes place during the dry season, which is therefore the focus of this study.

Observational data reveal changes in precipitation patterns since the middle of the past century, with some regions in the world getting increased precipitation while other regions are experiencing less precipitation^18^,^19^. In general, increases in anthropogenic emissions have led to more precipitation on a global scale due to surface temperature warming^20^. However, the detailed effects of changes in greenhouse gases and aerosols on the hydrological cycle are complex^2^,^21^. Precipitation responses depend on the climate forcing mechanism, and also differ with timescale^15^,^16^,^22^. Responses in precipitation often show strong regional differences. This is mainly due to large-scale changes in circulation patterns and land-ocean contrasts, but could also arise from strictly local interactions, which are often caused by constituents that have short residence times in the atmosphere, such as BC.

Southern Africa is a region where observations indicate reduced precipitation and increased dryness since the 1950s (refs 18, 19). The region is highly vulnerable to climate change (see ref. 23 and references therein), and has strong connections between climate and water, energy and food security^24^. Global climate models predict a further decrease in precipitation and a strong increase in the number of consecutive dry days near the end of the century for a business-as-usual scenario, due to widening of the Hadley Circulation^25^. While previous work on observed precipitation has focused mostly on annual trends, the focus in this study is on the dry season during which emissions from biomass burning are particularly strong. Although long-term observational data are sparse in the region, previous studies have shown evidence of declining trends in annual precipitation for several stations. In the latter half of the 20th century, significant declining precipitation trends have been shown in Zambia and southwest Tanzania, in addition to a more widespread increase in the annual maximum of consecutive dry days^26^. Annual precipitation has also decreased in tropical rainforest regions, such as in Congo, the Democratic Republic of the Congo and Gabon^27^,^28^.

The present study focuses on explaining causes of observed precipitation decline during the dry season in southern Africa. We use a global precipitation observation data set (Global Precipitation Climatology Centre (GPCC)^29^,^30^) and a global (CESM1.0.4/CAM4 (refs 31, 32)) and regional climate model (Weather Research and Forecasting (WRF)^33^). Our results show that the combination of BC/OC from local anthropogenic biomass burning activities, and increases in the global CO_2_ concentration, can explain the precipitation decline in the region. These findings are further supported by analysis of the regional energy budget, which shows a local drying of the atmosphere from added biomass burning aerosols.

## Results

### Spatial distributions of precipitation changes

Some of the largest BC and OC emissions in the world take place in southern Africa due to extensive biomass burning during the dry season^34^,^35^, approximately from June to September each year. Due to human activities, BC biomass burning emissions in the region almost doubled during the 20th century, with the majority of the increase in the latter half^35^ (Supplementary Fig. 1), leading to a 60% increase in the BC burden^36^ (Table 1). The increase in OC burden over the period is about 70% (Table 1).

In the present study, we show the modelled precipitation impact of increases in local BC and OC emissions due to biomass burning over the past one-and-a-half centuries, and compare them with the possible influence of increases in atmospheric CO_2_ concentrations over the same period. We quantify and interpret the results from global and regional model results in terms of the regional energy budget^37^ to uncover the mechanisms behind modelled changes in precipitation.

Throughout the manuscript, we show differences between model experiments (see Table 1) and denote ‘BB' as the impact of changes in biomass burning emissions of BC and OC from 1850 to 2000 (experiments BASE−BB1850), ‘CO2' as the impact of changes in CO_2_ volume mixing ratios over the same time period (BASE−CO21850) and ‘CO2BB' as the combined impact of BC/OC biomass burning and CO_2_ concentration changes (BASE−CO2BB1850). Due to large variability in the precipitation changes, five-member ensemble runs were performed for each scenario.

**Figure 1:**
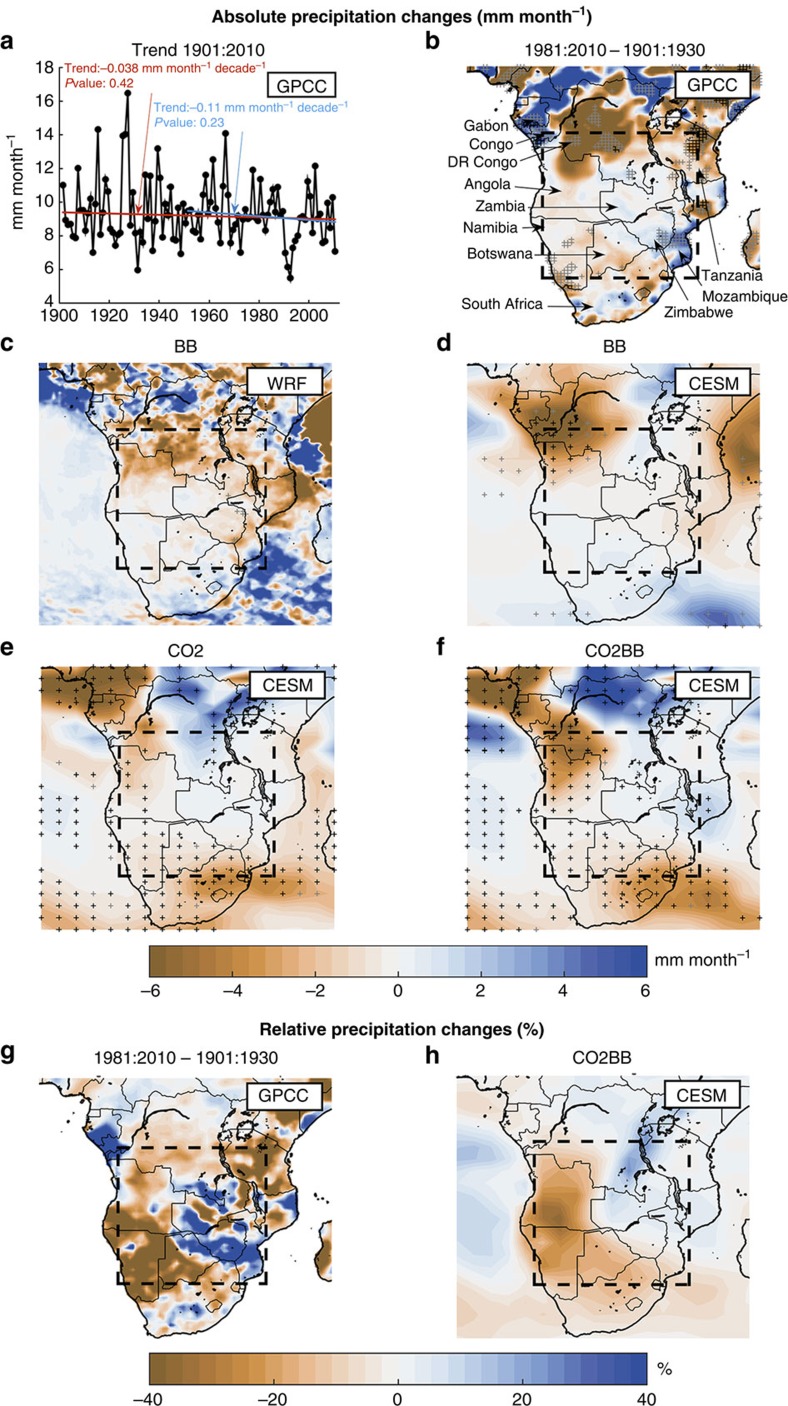
Distributions of observed and modelled precipitation changes. Trend in observed precipitation (mm month^−1^) over the June to September season (**a**), absolute precipitation changes (mm month^−1^) for the same season from observations and various model experiments (**b**–**f**), and relative precipitation changes (%) from observations and the CO2BB model experiment (**g**–**h**). Values in (**a**) are averaged over the subregion shown by the dashed rectangle in (**b**). Panels **a**,**b** and **g** are from observations (GPCC) whereas the five other panels are from models results, one with the regional WRF model (**c**) and four from the global CESM model (**d**–**f**,**h**). Model results are shown as differences from the BASE simulation and show differences between 2000 and 1850 conditions in each case; see text and Table 1 for notation of the experiments. Observed precipitation trends in (**a**) are shown for the whole GPCC time period from 1901 to 2010 (red) and from 1951 to 2010 (blue). Observations in (**b**) are shown as the difference 1981:2010−1901:1930 because 30-year averages are needed to reduce noise (that is, the influence of natural variability). Note that in most parts of the region, the number of stations per grid cell is low in the GPCC data. In plots (**b**–**f**), symbols denote grid boxes where changes are significant (*P* value <0.05) according to a two-tailed Student's *t*-test (grey ‘+' symbols), and when also accounting for multiple statistical testing according to ref. 54 (black ‘+' symbols).

Observations over southern Africa in the past century indicate a precipitation decline during the dry season from June to September (Fig. 1a,b), with a stronger decline in the latter part of the period, coinciding with the period of strongly increasing biomass burning emissions (Supplementary Fig. 1b).

A strong impact of anthropogenic biomass burning particles on precipitation during the dry season can be seen in the regional and the global climate model (Fig. 1c,d; Supplementary Fig. 2c,d), with precipitation decreases in the order of 10–40% in large parts of the region. In both models the strongest decline in absolute precipitation occurs in the northwest, which also is the region with the largest increase in atmospheric biomass burning aerosol burden (Supplementary Fig. 1c). In this region, the global (Community Earth System Model (CESM)) model BB and CO2BB simulations show statistically significant reductions in precipitation. The BB simulation in the regional (WRF) model supports this finding, showing precipitation reduction in the same area, although not statistically significant due to a smaller sample of years. An overall decrease in precipitation can be seen in southern Africa, but with some smaller regions experiencing increased precipitation due to biomass burning. Such regions of increased precipitation downwind of regions where BB leads to precipitation decreases can be expected and have been seen before^11^. The regional model, with higher spatial resolution than the global model, shows a more inhomogeneous distribution with more local scale features.

The modelled impact of industrial-era changes in CO_2_ concentrations on precipitation is an increase in the northeast of southern Africa and a decrease of up to 30% in most of the remaining part (Fig. 1e; Supplementary Fig. 2e). Thus, our results indicate that the known changes in both BB and CO_2_ concentrations may have led to a precipitation decrease since 1850, but with slightly different patterns. This decrease is enhanced in the simulation with combined changes in BB and CO_2_ concentrations (Fig. 1f,h). The precipitation pattern in the subregion in CO2BB resembles that of the sum of BB and CO2 (not shown), indicating that the two effects are to a large extent additive. Distributions of changes in the number of dry days (that is, days with precipitation <1 mm) and maximum number of consecutive dry days show similar tendencies of a drying due to BB and increased CO_2_ concentrations (Supplementary Figs 3 and 4).

Modelled precipitation changes compare relatively well with what has been observed over the previous century (Fig. 1; Supplementary Fig. 2). Notably, observed precipitation decreases close to regions where biomass burning emissions have increased most are well reproduced by both models' BB experiment, particularly in Angola and south of the Democratic Republic of the Congo. Correlation coefficients for the subregion confirm that observed spatial patterns of absolute precipitation change correlate well with modelled precipitation changes induced by increased biomass burning (correlation coefficients >0.5 at 2.5° resolution, and significant at *P*<0.05 level), while there is no correlation with modelled precipitation change due to increased CO_2_ (correlation coefficient ∼0.0) (Supplementary Table 1). Uncertainties usually increase when going from larger to smaller scales in studies of climate change impacts, and a small displacement in simulated patterns may therefore lead to poor correlation. We note here that correlation coefficients calculated after averaging to coarser resolution are much higher (for example, 0.7–0.8 for BB at 5° resolution). It is also encouraging that the models are able to reproduce the spatial patterns of present-day precipitation climatology, with significant (*P* value<0.05) correlation coefficients of 0.97 and 0.78 for WRF and CESM, respectively (Supplementary Fig. 5; Supplementary Table 1).

The domination of BB aerosols in driving precipitation changes is unique for the dry season in the southern Africa region. Anthropogenic biomass burning aerosols are not found to dominate precipitation changes in other regions or seasons. In central Africa, biomass burning activities mainly take place from December to February, but the increase in biomass burning aerosol emissions since pre-industrial times has not led to a clear impact on precipitation (Supplementary Fig. 6). The third region of the world, in addition to southern and central Africa, with strong increases in biomass burning activities is the central part of South America. Here observations indicate a precipitation decline during its dry season from June to September, and global model results show that this is caused by changes in CO_2_ rather than changes in BB (Supplementary Fig. 7).

### Changes in mean precipitation and number of dry days

In the following we focus on the southern African subregion indicated by the dashed rectangle in Fig. 1. This region has had a strong increase in biomass burning emissions, and is a region with minimal precipitation during the June to September period, implying that a climatic change in precipitation could have important societal effects.

**Figure 2:**
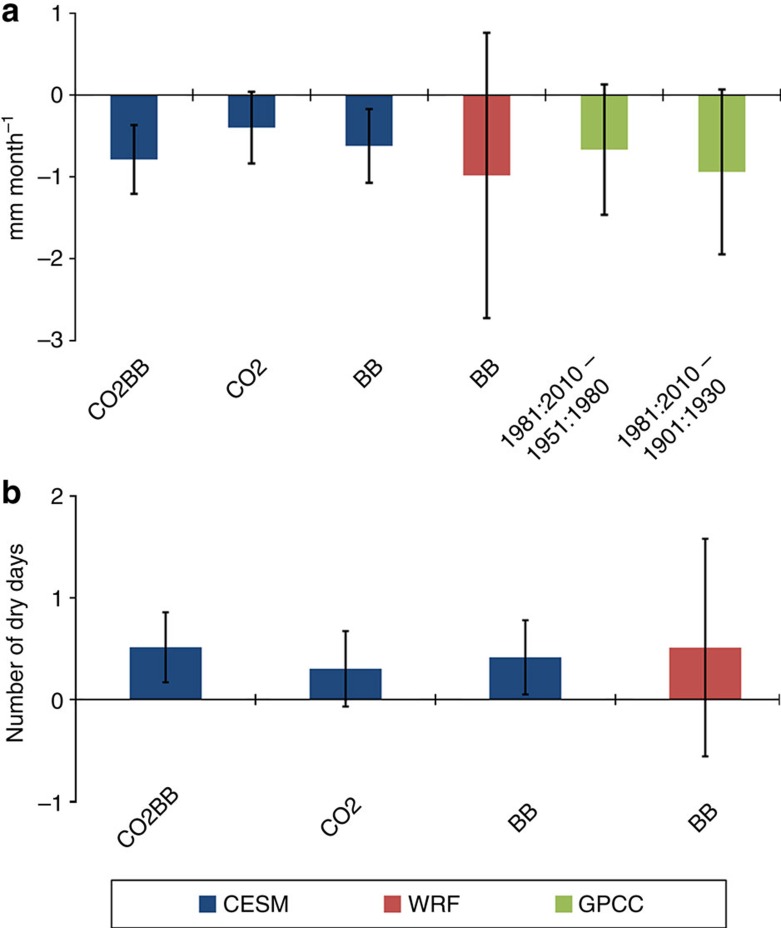
Southern Africa average change in precipitation. Change in mean precipitation (mm month^−1^) (**a**) and number of dry days (**b**) over the June to September season within the subregion (region indicated by dashed rectangle in Fig. 1) for various model experiments. Model results are shown as differences from the BASE simulation, where CESM denotes the global climate model and WRF the regional model. GPCC observations are both shown as the difference 1981:2010−1901:1930 and 1981:2010−1951:1980. Error bars show the 95% confidence interval derived from a two-tailed Student's *t*-test.

The reduction in precipitation in the subregion due to BB and anthropogenic CO_2_ emissions is evident from Fig. 2, with a decrease in monthly mean precipitation and an increase in the number of dry days. This precipitation reduction is also observed, and although the absolute precipitation change of around 1 mm month^−1^ seems small, the relative change is as much as 5–10% (Supplementary Fig. 8). It is also an average over a relatively large area, locally observed changes are much larger (Fig. 1b,g). With the exception of one ensemble member for the BB case and one for the CO2BB case, the decrease in mean precipitation and increase in the number of dry days are consistent for all cases, between all ensemble members, and across the two models (Fig. 2; Supplementary Fig. 8). In the BB and CO2BB cases, the results are statistically significant (*P* value<0.05) for all ensemble members aggregated (Fig. 2). The WRF model result is based on fewer years and therefore not statistically significant at the same level, but is still in general agreement with the mean of the CESM ensembles.

### Precipitation and the regional atmospheric energy budget

Analysis of the energy budget, including changes in horizontal energy transport, have proved useful in understanding global and regional precipitation changes^21^,^37^. The local atmospheric energy budget representing the difference between two climate situations can be approximated by


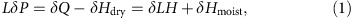


where *L* is the latent heat of condensation, *δP* is the change in surface precipitation flux, *δQ* is the change in diabatic cooling of the atmosphere, *δH*_dry_ and *δH*_moist_ denote the change in net inward horizontal dry and moist static energy transport, respectively, and *δLH* is the net upward latent heat flux from the surface (see Methods section and refs 21, 37 for details). The diabatic cooling term (*δQ*) can be written *δQ*=−(*δSW*+*δLW*+*δSH*), where *δSW* and *δLW* is the net atmospheric shortwave and longwave radiative heating, respectively, and *δSH* is the upward sensible heat flux from the surface. Equation 1 shows that precipitation changes are constrained by the atmospheric energy balance^21^.

**Figure 3:**
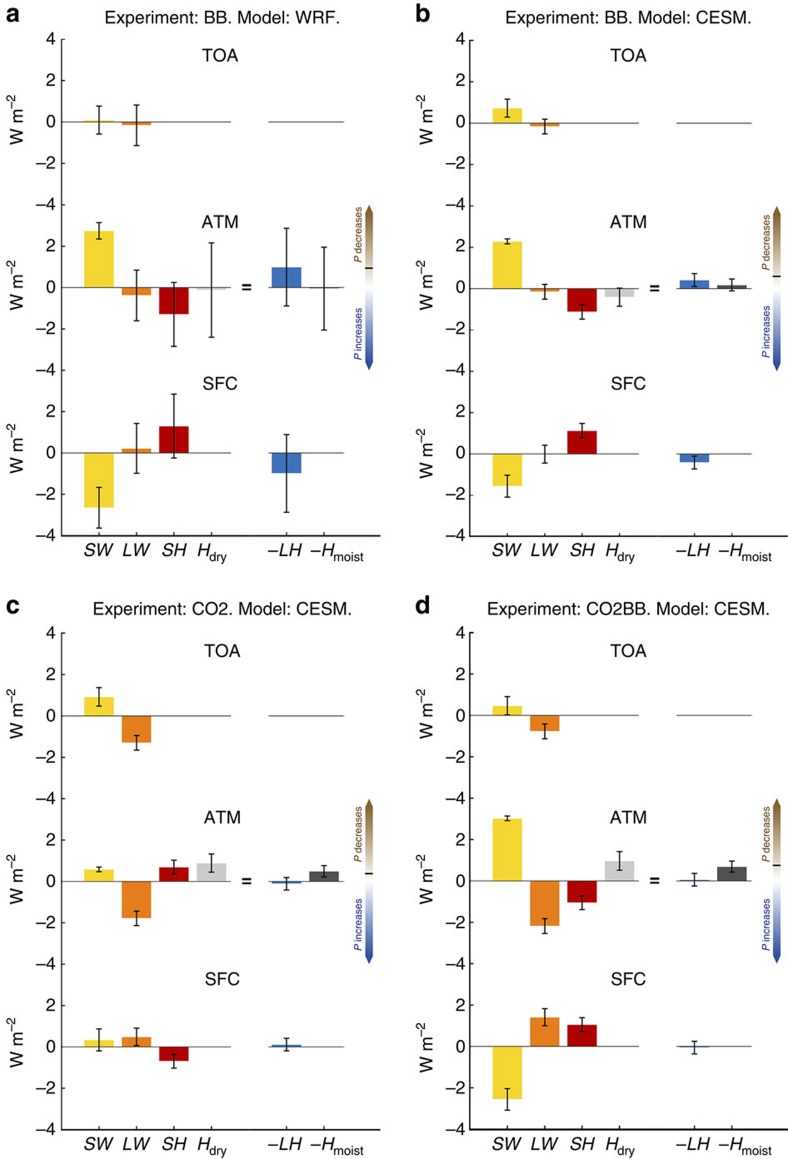
Regional atmospheric energy budget for southern Africa. Atmospheric energy budget over the June to September season and averaged over the subregion (shown by the dashed rectangle in Fig. 1) for different models and experiments. Results are shown as differences from the BASE simulation, where CESM denotes the global climate model and WRF the regional model. At top-of-atmosphere (TOA) and surface (SFC), net downward energy fluxes have positive values. The energy budget of the atmosphere (ATM) is defined as the difference between the energy balance at TOA and SFC, except for the horizontal transport terms (*H*_dry_ and *H*_moist_), which are positive when net energy transport is into the atmospheric column from the outside. Direction of brown and blue arrows on the right-hand-side of each plot denotes contribution towards decreased or increased surface precipitation according to equation 1 (−*LδP*=*δSW*+*δLW*+*δSH*+*δH*_dry_=−*δLH*−*δH*_moist_), and the total change in precipitation in each experiment is indicated by a black line embedded on these arrows. Error bars show the 95% confidence interval derived from a two-tailed Student's *t*-test.

Regional energy budgets for each model experiment are shown in Fig. 3 and Supplementary Fig. 9. Net atmospheric absorption of solar radiation from added BC in southern Africa is strong and exceeds 2 W m^−2^ (Fig. 3a,b). The level of agreement between the two models and between the various ensemble members is high relative to the other terms that experience much larger natural year-to-year variability.

The atmospheric radiative heating due to solar absorption by BC, or in other words, the reduced diabatic cooling term (*δQ*) caused by BC, leads to a decrease in surface precipitation flux (assuming *L* is constant). However, this is partly balanced by reduced sensible heat flux from the surface to the atmosphere caused by reduced solar radiation reaching the surface. Scattering by OC particles also contributes to the reduced sunlight at the surface in the BB case. Absorbing aerosols have previously been shown to cause circulation changes, due to the induced temperature gradients, and this depends principally on the vertical location and magnitude of the aerosols^38^. Here, in the global model, increased net horizontal transport of dry static energy outward from the atmospheric column, presumably resulting from circulation changes, draws in the same direction as the sensible heat term (Fig. 3b). In the regional model, the horizontal transport terms are negligible (Fig. 3a), and this difference seems to be the main reason for the larger precipitation reduction in the regional model, compared with the global model, seen in the BB case in Fig. 2a.

The local energy budget for increased CO_2_ concentrations is fundamentally different from that for the BB case (Fig. 3c). Increased CO_2_ leads to increasing global sea surface temperatures, thus feedback effects dominate the atmospheric energy budget. The main change in the local atmospheric energy budget is from the LW radiative cooling term, caused by increased surface temperature, partly offset by atmospheric absorption^39^, and contributes to increased precipitation. However, this precipitation increase is counterbalanced by increases in atmospheric SW heating caused mainly by increased solar absorption by water vapour^37^, sensible heat flux from the surface, and net inward horizontal energy flux due to large scale circulation changes. Increase in CO_2_ leads to a precipitation decline; despite a small increase in latent heat flux from the surface to the atmosphere, a net loss of moist static energy in the horizontal direction leads to an overall decrease. Although the effect of increased CO_2_ concentrations is a precipitation increase on a global scale, this local decrease in precipitation is not unexpected as the spatial pattern of precipitation changes due to anthropogenic climate change is to a large extent affected by circulation changes^37^. In fact, analysis of modelled vertical velocity shows a widening of the Hadley cell in the increased CO_2_ case, consistent with what has been observed^40^. This widening leads to more subsidence, and presumably less precipitation, south of ∼18°S in the subregion, and slightly less subsidence north of this latitude (not shown), largely consistent with modelled precipitation changes (Fig. 1e; Supplementary Fig. 2e).

When both adding biomass burning aerosols and increasing CO_2_, the decrease in precipitation is driven by shortwave atmospheric absorption and horizontal transport of both dry and moist static energy (Fig. 3d). Overall the regional energy budget explains the precipitation changes illustrated in Figs 1 and 2, with dissimilar impact on the energy budget from CO_2_ and BB. Comparison of an additional transient simulation that includes changes in BC/OC from biomass burning and increased CO_2_ concentrations, and a transient simulation including all anthropogenic climate forcers, indicate that increases in CO_2_ and biomass burning BC/OC are the main drivers of changes to the atmospheric energy budget since pre-industrial time (Supplementary Fig. 10).

## Discussion

It should be noted that long-term measurements of precipitation in most of southern Africa are sparse^23^, and different observed precipitation products show relatively large discrepancies^41^. There may also be other drivers additional to BB and CO_2_, such as non-CO_2_ greenhouse gases and other aerosols, which may have contributed to the observed precipitation changes. However, reflective aerosols such as sulphate are not likely to contribute substantially because emissions of its main precursor, SO_2_, are relatively small in and around the surrounding region^35^. Furthermore, remote influences from the mid-latitudes cause only small effects in southern Africa^42^.

Previous studies have also found a clear impact of biomass burning aerosols on the hydrological cycle over southern and central Africa^5^,^11^,^43^,^44^. Although different experimental setups and focuses make it difficult to compare, there are some contradictory results in previous studies; some studies indicate more precipitation^5^,^43^ from biomass burning aerosols and others indicate less precipitation^11^,^44^, as is found here. Impacts of BC on clouds and precipitation are known to depend strongly on the altitude of added BC^3^,^14^, and this could be one reason for the differing results. The BC and OC aerosol concentrations in this study are from a model that includes a plume rise assumption for biomass burning emissions (OsloCTM2), and this leads to a portion of the aerosols being emitted above the boundary layer. Consequently, the main atmospheric heating from biomass burning BC aerosols occurs at an altitude high enough to inhibit vertical instability under certain conditions, and biomass burning BC may therefore suppress convective activity in our simulations. While we acknowledge that uncertainties in BC vertical distribution are large, it is well established that biomass burning emissions are rapidly lifted to high altitudes^45^. OsloCTM2 vertical profiles of extinction coefficients compare very well with the CALIOP satellite instrument over the southern Africa region (see Fig. 4 in ref. 46).

Although the findings in this study are supported by a regional and a global model, observations, and analysis of the regional energy budget, uncertainties exist when studying changes in precipitation on a regional scale. A multi-model study would be required to provide further robustness to the findings.

The global climate model calculations in the present study were performed using a slab ocean setup, to reduce the required computational resources. We do not expect that our results would differ largely if a full ocean rather than a slab ocean model had been used. In a study using an earlier version of the CESM model^47^, a fully coupled ocean model simulation led to a slightly higher climate sensitivity. Hence, a slightly stronger response in the hydrological cycle could possibly have been expected in the increased CO_2_ case if a full ocean model would have been used. The impact of BC on the hydrological cycle is however dominated by rapid adjustments^15^ and is therefore not likely to be strongly affected by the choice of slab ocean versus full ocean.

Our results show that local BC and OC aerosol emissions from anthropogenic biomass burning activities and increasing global CO_2_ concentrations are the main causes of the observed decline in southern African dry season precipitation over the last century. This implies that, in addition to improving air quality, reducing biomass burning emissions of BC and OC may be a useful way to mitigate reduced rainfall in the southern Africa region. This conclusion is strengthened by the suggestion that these aerosols may be part of a positive feedback loop where increased drought could increase the risk of igniting more fires^11^. Precipitation in the region is expected to decrease further in the future if greenhouse gas emissions continue unabated^25^. Hence, initiation of stringent air quality legislation, involving BC mitigation, may help to counteract this precipitation decline in the short term.

## Methods

### Observation data

We have used long-term observation data from the GPCC^29^,^30^, which provides monthly values of precipitation from 1901 to 2010 from 67,200 stations world-wide, gridded to a horizontal resolution of 0.5° × 0.5°. This observation dataset has been chosen because it gives a better coverage than other long-term gridded precipitation observation datasets, such as CRU and GHCN – see TFE.1, Fig. 2 in ref. 48. In regions where GPCC coverage overlaps with CRU and GHCN, they give similar estimates of annual trends. The trend analysis in Fig. 1a has been calculated using the Mann–Kendall method^49^, and *P* values in the observed trends are calculated using a two-tailed *z*-test.

### Global climate model

Global climate model simulations have been carried out using the National Center for Atmospheric Research (NCAR) CESM1.0.4 (ref. 31). The atmospheric component, Community Atmosphere Model (CAM4) (ref. 32), was in our simulations run with 1.9° × 2.5° horizontal resolution and 26 vertical layers, and coupled with the Community Land Model (CLM4) (ref. 50). Five different ensemble members have been run for 60 years each, whereof the first 10 years were considered as spin-up and are not part of the analysis. The ensemble members were started from different initial conditions but the same climatological state, and we have used a configuration where the atmospheric component is coupled with a slab ocean model^51^. Results are shown as averages of all ensemble members. Atmospheric distributions of aerosol concentrations have been prescribed for black and organic carbon, and sulphate, using monthly mean 3-D concentrations from the chemistry-transport model OsloCTM2 from a previous study^36^. This model has previously shown good agreement with observed pattern and magnitude of aerosol optical depth in southern Africa during the biomass burning season^34^, and with vertical aerosol extinction coefficient profiles over the same region^46^. In the CESM model experiments, both the CO_2_ and BC/OC concentrations have been changed globally. To test the effect of global versus local perturbation of biomass burning BC/OC, the first two ensemble members of the BB1850 simulation were re-run, but with biomass burning BC/OC concentrations changed to 1850 levels only during June to September and only in and around southern Africa. The results in terms of southern Africa dry season precipitation change were within 25% of the results obtained when biomass burning BC/OC were changed globally and annually.

### Regional climate model

Additional climate model simulations have been conducted using the regional WRF model^33^ version 3.5. The model has been run in 50 × 50 km horizontal resolution over Africa, and with 50 vertical layers from the surface and up to 10 hPa. The extent of the horizontal domain is the same as defined in CORDEX-Africa^52^. WRF has been run for the 30 year period, 1984–2013, in 1 year time slices, each re-initialized in December of the previous year to allow one month of spin-up. Meteorological initial and boundary conditions, as well as sea-surface temperatures, have been taken from the 6-hourly 1° × 1° resolution final reanalysis data of the Global Forecast System (GFS) model of the National Centers for Environmental Prediction (NCEP) from year 2000 onward, and NCEP/NCAR 2.5° × 2.5° resolution reanalysis for the preceding years^53^. The same radiation scheme (CAM) as in the CESM has been used in WRF. In the standard version of WRF, however, the CAM scheme only allows one fixed column value of each aerosol species to represent the entire domain for the whole year. Here, the WRF code was modified to enable prescribed monthly mean 3-D concentration fields of BC, organic carbon and sulphate from OsloCTM2, for consistency with what is used in the CESM.

### Energy budget

In the calculation of *δH*_dry_, we have used the same approach as in ref. 37 and calculated it as the residual of equation 1 (*δH*_dry_=*LδP*−*δQ*), assuming *L*=29 W m^−2^ mm^−1^ day, rather than a direct calculation of the total dry static energy flux. This approximation has been shown to introduce only small errors^37^. In Fig. 3, we have represented differences in horizontal transport of energy in and out of the atmospheric region both as dry static energy flux (*δH*_dry_) and as moist static energy flux (*δH*_moist_). The latter term is again calculated as a residual, *δH*_moist_=*δLH*-*LδP*, and both *LH* and *P* are available from the model output.

## Additional information

**How to cite this article:** Hodnebrog, Ø. *et al.* Local biomass burning is a dominant cause of the observed precipitation reduction in southern Africa. *Nat. Commun.* 7:11236 doi: 10.1038/ncomms11236 (2016).

## Supplementary Material

Supplementary Information Supplementary Figures 1-10, Supplementary Table 1 and Supplementary References.

## Figures and Tables

**Table 1:** Overview of model experiments.

Experiment*	**Short description**	**Global CO**_**2**_ **vmr (p.p.m.)**	**BC burden**† **(mg m**^−**2**^**)**	**OC burden**† **(mg m**^−**2**^**)**
BASE	Year 2000 conditions	367	1.85	11.8
BB1850	Biomass burning emissions of BC/OC for 1850	367	1.15	6.79
CO21850	CO_2_ concentrations for 1850	285	1.85	11.8
CO2BB1850	Both CO_2_ and BB changed to 1850	285	1.15	6.79

^*^BB=BASE−BB1850; CO_2_=BASE−CO21850; CO2BB=BASE−CO2BB1850.

^†^Burdens are averaged over southern Africa (subregion shown by dashed rectangle in Fig. 1) and over the June to September season.
