# Supplementary material for: Local biomass burning is a dominant cause of the observed precipitation reduction in southern Africa
Source: Nat Commun. 2016 Apr 12;7:11236. doi: 10.1038/ncomms11236 (PMC4832063; doi:10.1038/ncomms11236)
Supplement: Supplementary Information — Supplementary Figures 1-10, Supplementary Table 1 and Supplementary References. [file ncomms11236-s1.pdf]

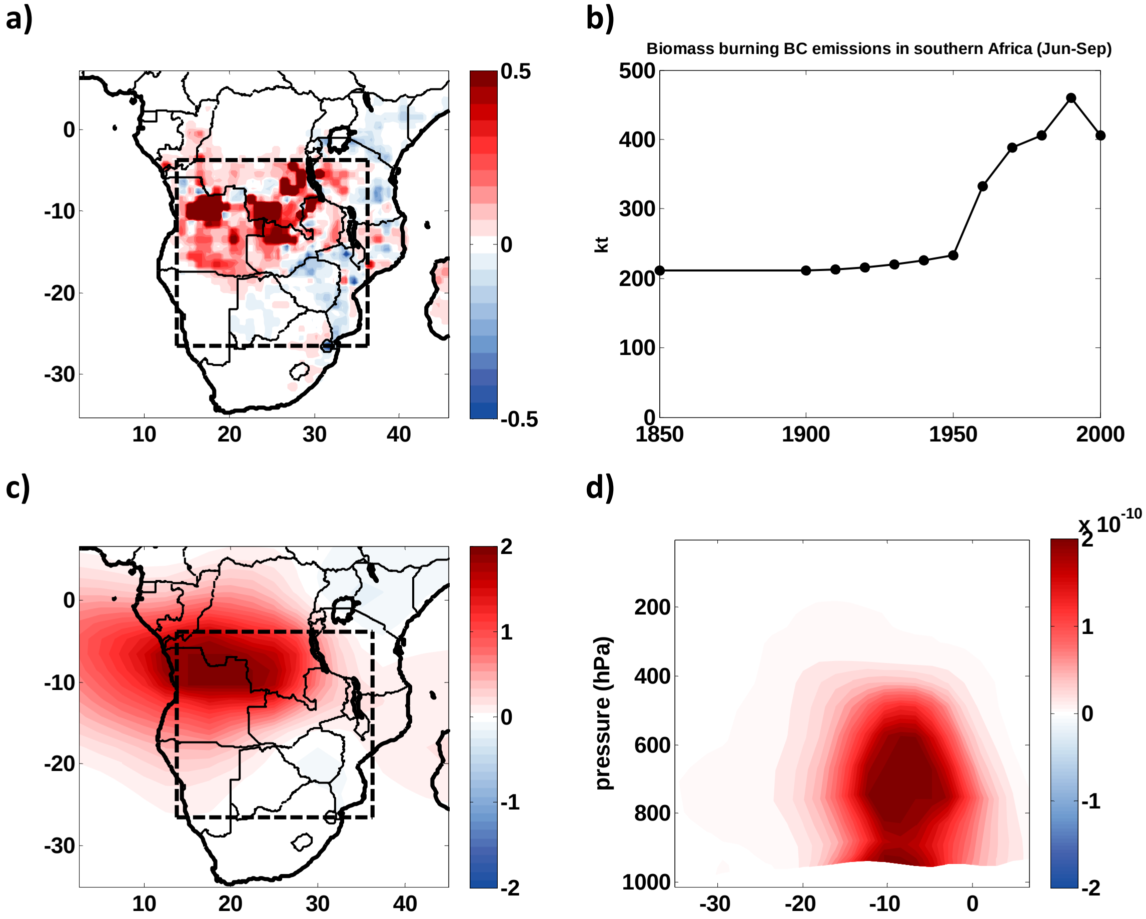

Supplementary Figure 1. Emissions and atmospheric concentrations. Change in biomass burning emissions (kilotonnes) of black carbon (BC) from 1850 to 2000 **(a)**, time evolution of biomass burning BC emissions in the region shown by dashed rectangle in (a) **(b)**, difference in atmospheric column (mg m^{-2}) **(c)** and zonal mean (kg kg^{-1}) **(d)** of black carbon between the BASE and BB1850 simulations. All plots show data for the June to September season only. Emission data are from Lamarque et al.[1] and show a peak in 1990 as is the case globally (see their Table 9). Model data are from Skeie et al. [2]. The OC burden is not shown, but has a similar distribution as BC.

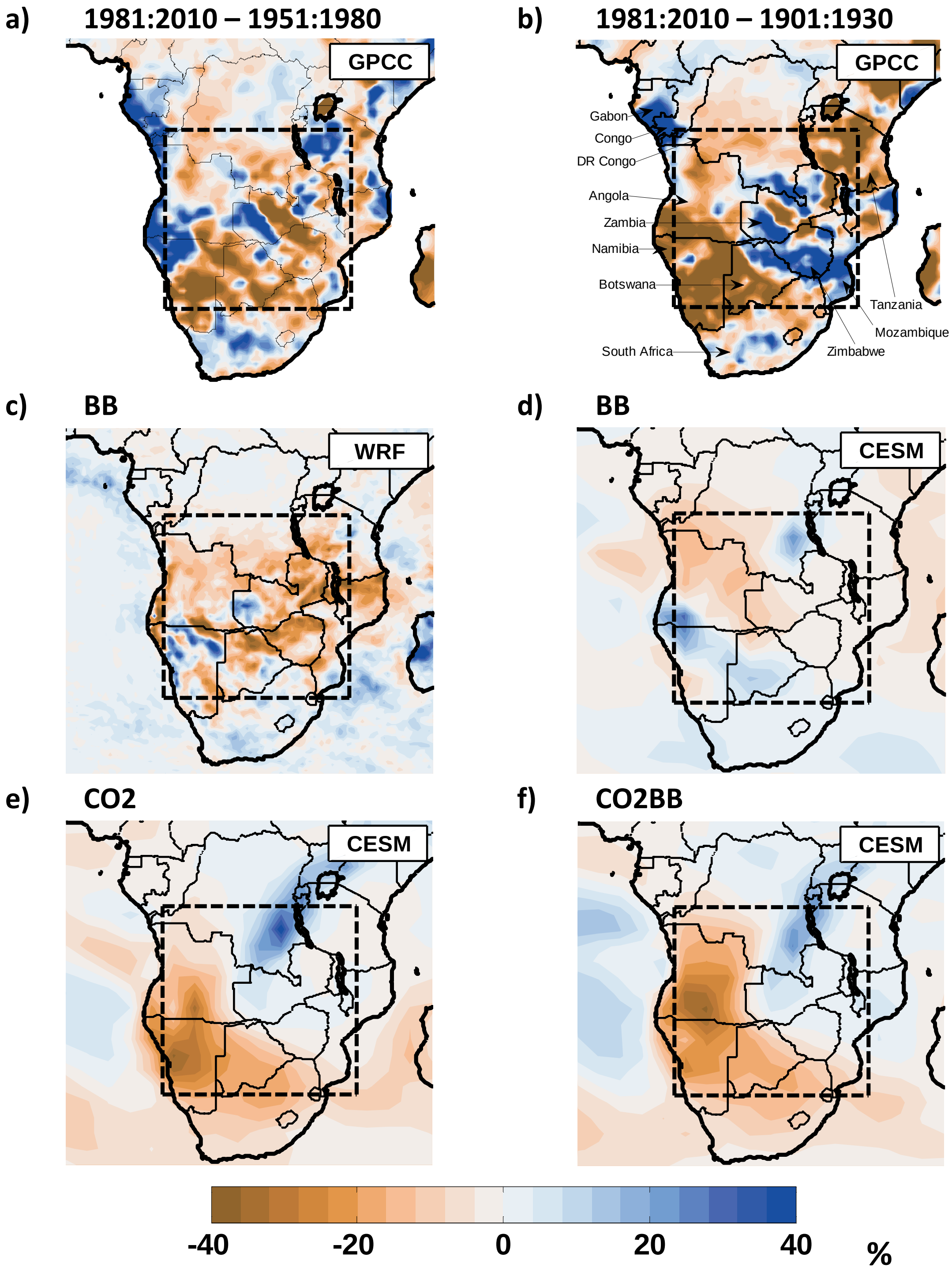

Supplementary Figure 2. Distributions of observed and modelled precipitation changes. Relative changes in precipitation (%) over the June to September season from observations **(a-b)** and various model experiments **(c-f)**. The top two panels (a-b) are from observations (GPCC) whereas the four other panels are from models results, one with the regional WRF model (c) and three from the global CESM model (d-f). Model results are shown as differences from the BASE simulation; see manuscript text and Table 1 for notation of the experiments. GPCC observations are shown as difference 1981:2010 – 1901:1930 and 1981:2010 – 1951:1980. Note that in most parts of the region, the number of stations per grid cell is low in the GPCC data.

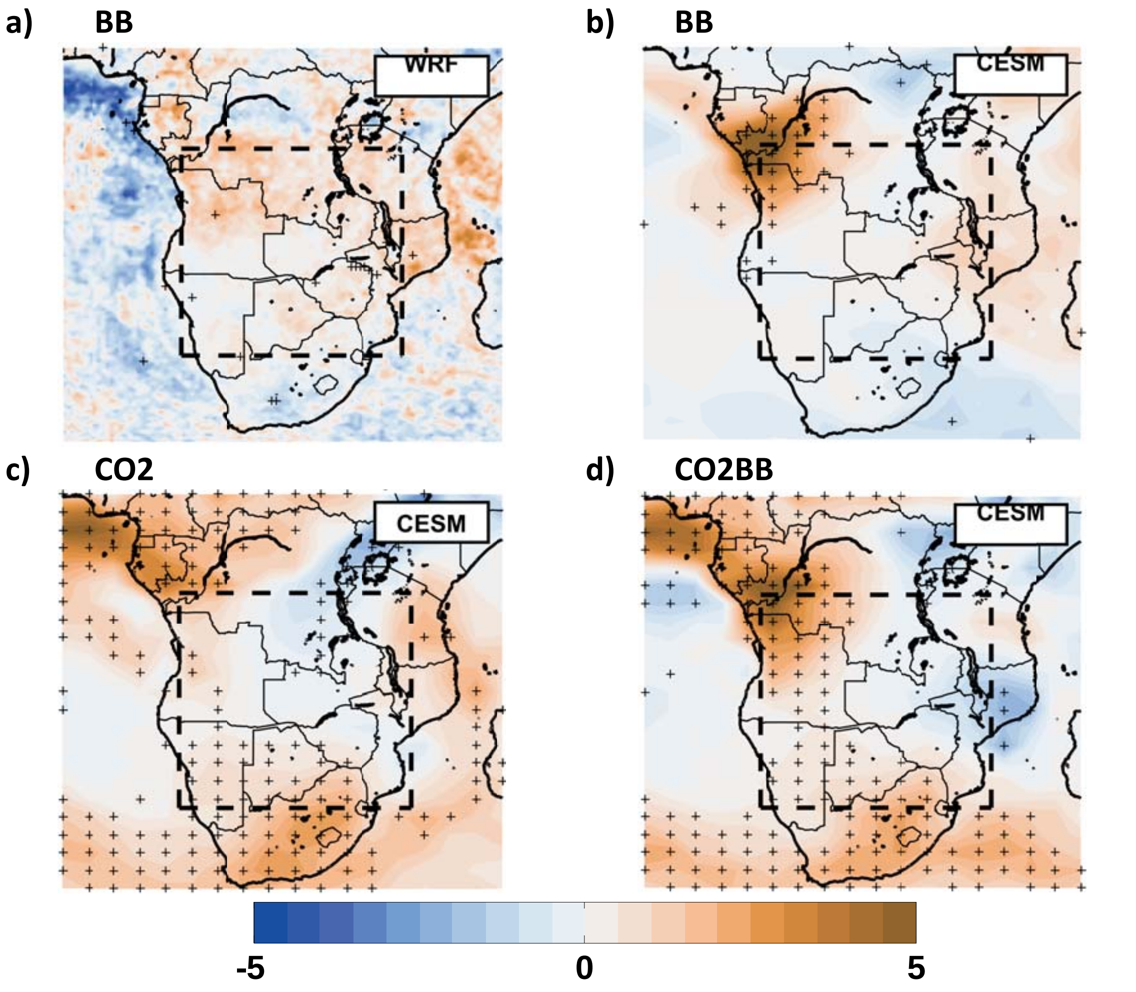

Supplementary Figure 3. Modelled changes in number of dry days. Change in number of dry days (days with precipitation less than 1 mm) during the June to September season (total of 122 days) for various model experiments. Model results are shown as differences from the BASE simulation; see manuscript text and Table 1 for notation of the experiments. The '+' symbol denotes grid boxes where changes are significant ($p < 0.05$) according to a two-tailed Student's *t*-test (multiple statistical testing is not accounted for).

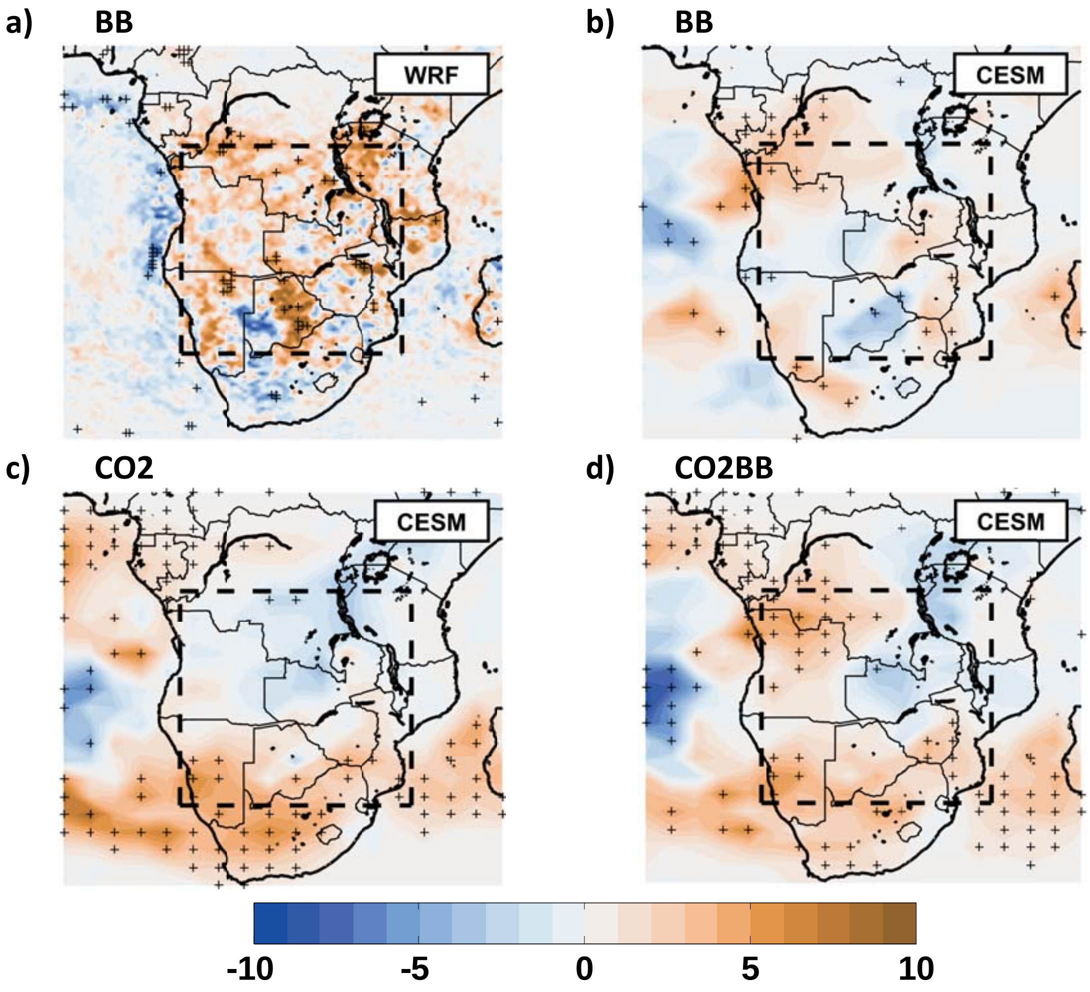

Supplementary Figure 4. Modelled changes in maximum number of consecutive dry days. Change in maximum number of consecutive dry days (days with precipitation less than 1 mm) during the June to September season (total of 122 days) for various model experiments. Model results are shown as differences from the BASE simulation; see manuscript text and Table 1 for notation of the experiments. The '+' symbol denotes grid boxes where changes are significant ($p < 0.05$) according to a two-tailed Student's *t*-test (multiple statistical testing is not accounted for).

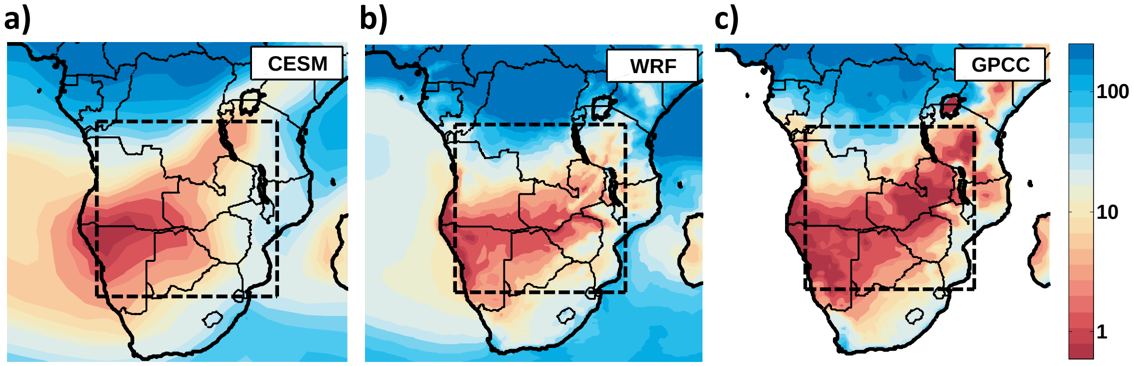

Supplementary Figure 5. Modelled and observed precipitation climatology. Comparison of precipitation (unit: mm month^{-1}) during June to September between the CESM **(a)** and WRF **(b)** models, and the GPCC observations **(c)**. CESM model results are averaged over 5 ensemble members of 50 year simulations using slab ocean configuration and year 2000 greenhouse gas and aerosol forcing, WRF model results are averaged over a 30 year fixed SST simulation using reanalysis data from 1984:2013 at the boundaries, while observations are averaged over 1981:2010. Note that only precipitation over land is shown in the observation data set.

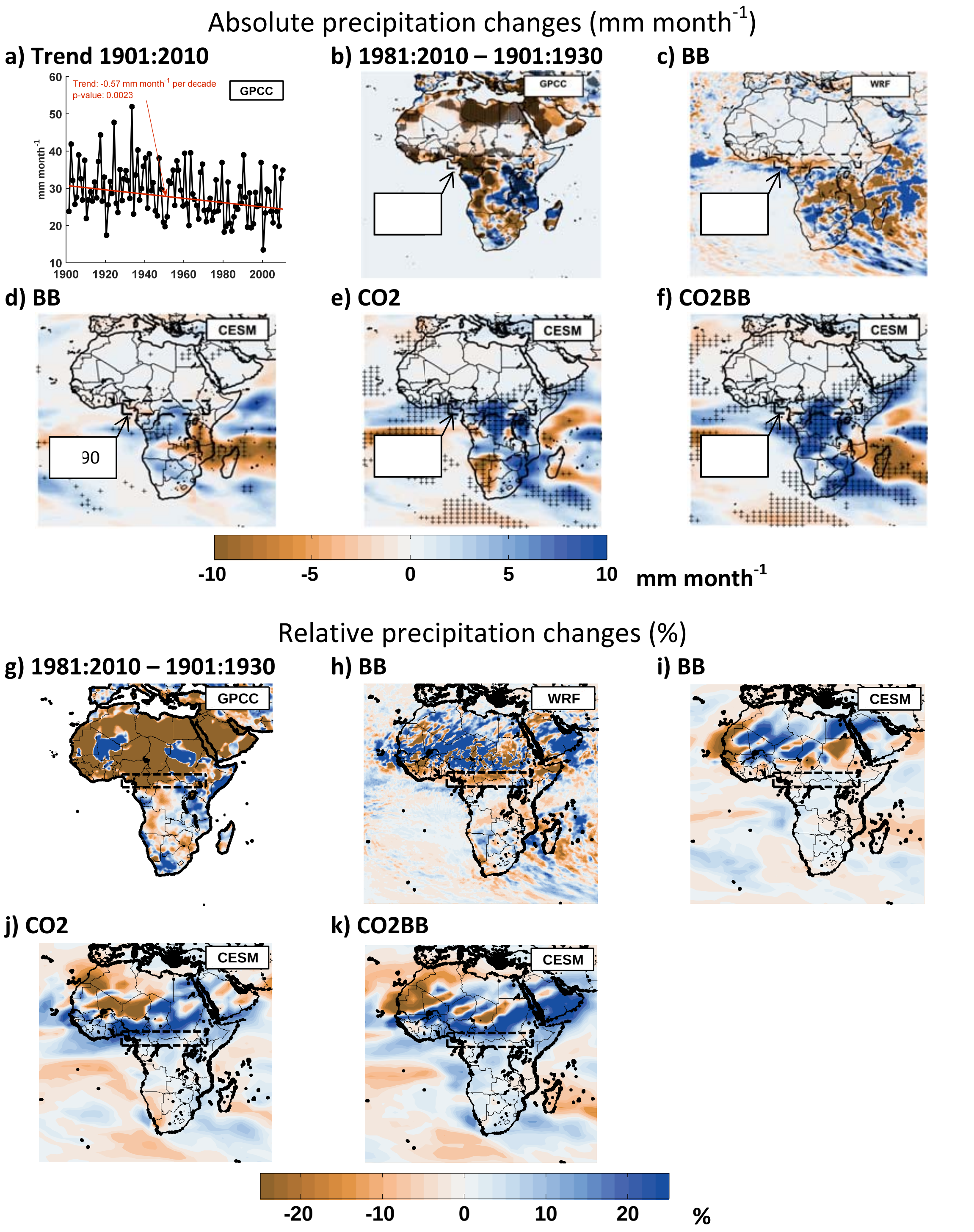

Supplementary Figure 6. Observed and modelled precipitation changes in Africa. Trend in observed precipitation (mm month^{-1}) over the December to February season **(a)**, absolute precipitation changes (mm month^{-1}) for the same season from observations and various model experiments **(b-f)**, and corresponding plots for relative precipitation changes (%) from observations and model experiments **(g-k)**. See text and Table 1 in the main manuscript for notation of the experiments and description of the models and observations. Values in (a) are averaged over the subregion shown by the dashed rectangle in (b). In plots (b-f), a '+' symbol denotes grid boxes where changes are significant ($p < 0.05$) according to a two-tailed Student's *t*-test (multiple statistical testing is not accounted for). The plots show that there is a link between BB and precipitation change for central Africa, but the net impact is relatively small and not consistent between the two models (WRF and CESM).

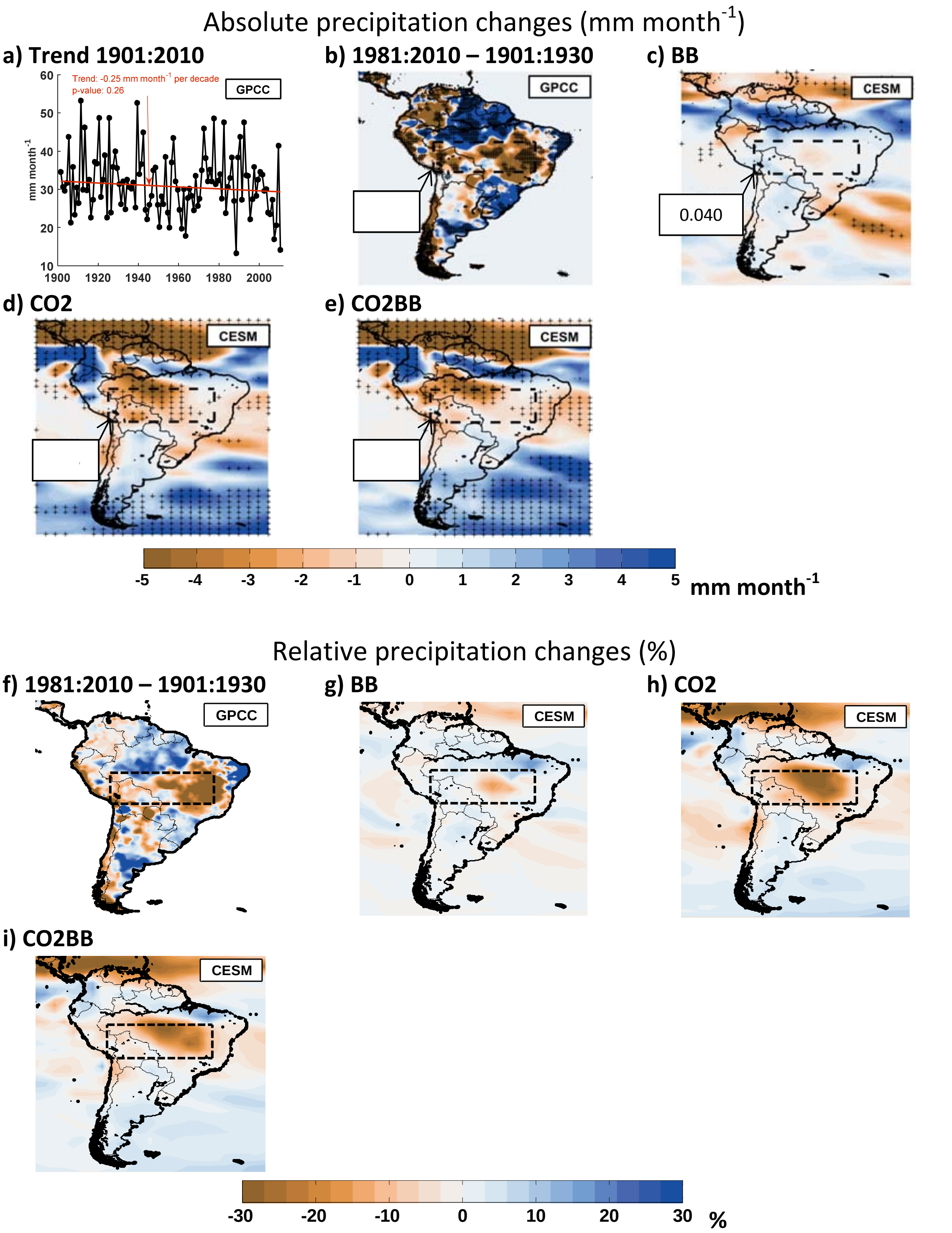

Supplementary Figure 7. Observed and modelled precipitation changes in South America. Trend in observed precipitation (mm month^{-1}) over the June to September season **(a)**, absolute precipitation changes (mm month^{-1}) for the same season from observations and various model experiments **(b-e)**, and corresponding plots for relative precipitation changes (%) from observations and model experiments **(f-i)**. See text and Table 1 in the main manuscript for notation of the experiments and description of the model and observations. Values in (a) are averaged over the subregion shown by the dashed rectangle in (b). In plots (b-e), a '+' symbol denotes grid boxes where changes are significant ($p < 0.05$) according to a two-tailed Student's *t*-test (multiple statistical testing is not accounted for). The plots show that the observed precipitation decrease in the subregion can more likely be explained by increases in CO_2 concentrations than by increased BB activity.

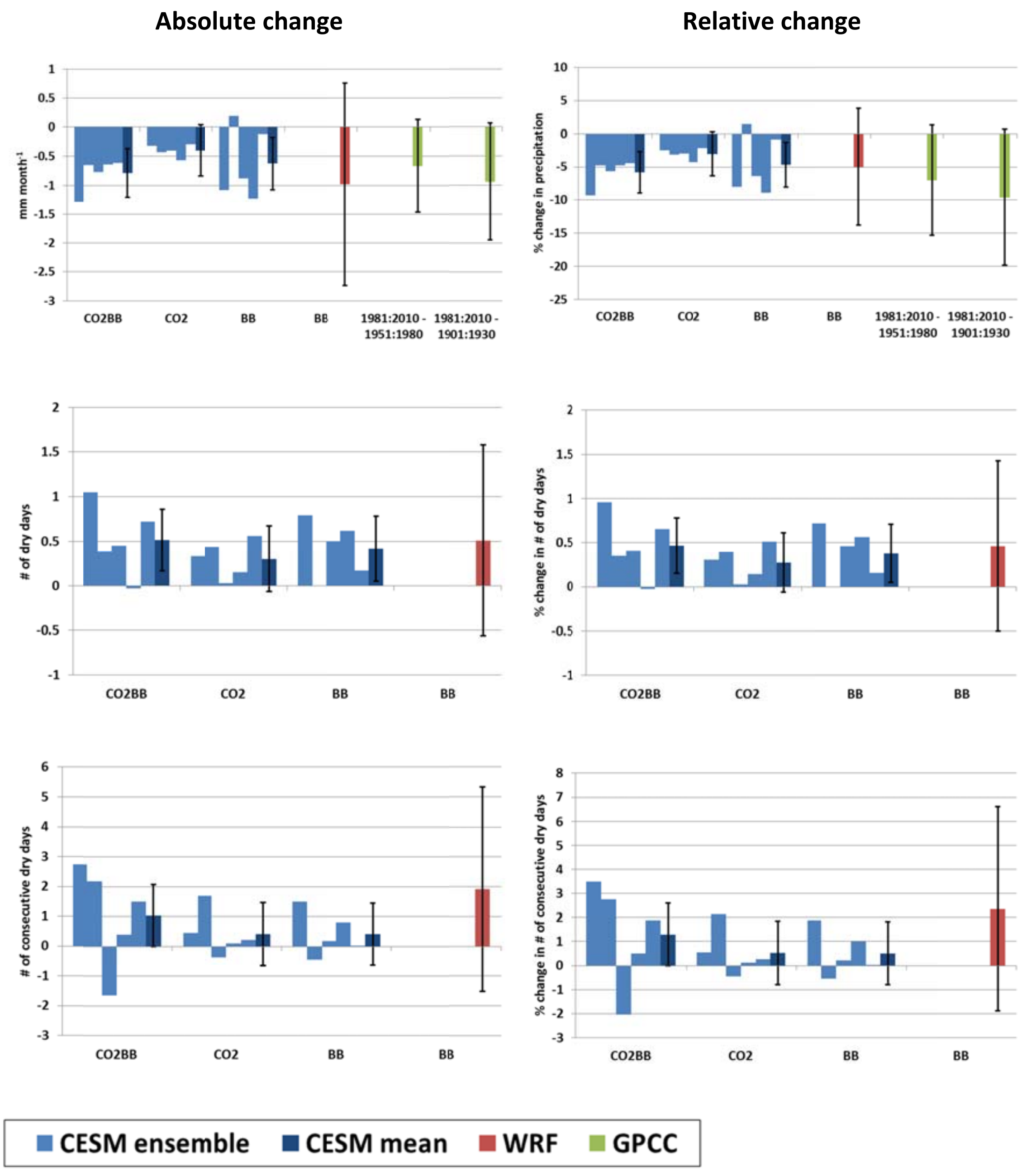

Supplementary Figure 8. Southern Africa average change in precipitation. Change in mean precipitation (mm month^{-1}; top panel), number of dry days (middle panel) and mean of the maximum number of consecutive dry days over the June to September season (total of 122 days) within the subregion (region indicated by dashed rectangle in Fig. 1) for various model experiments. Model results are shown as differences from the BASE simulation, where CESM denotes the global climate model and WRF the regional model. Relative changes (in %) are shown in the right column. GPCC observations are included in the top panel and are shown as the differences 1981:2010 – 1901:1930 and 1981:2010 – 1951:1980. Error bars show the 95% confidence interval derived from a two-tailed Student's *t*-test.

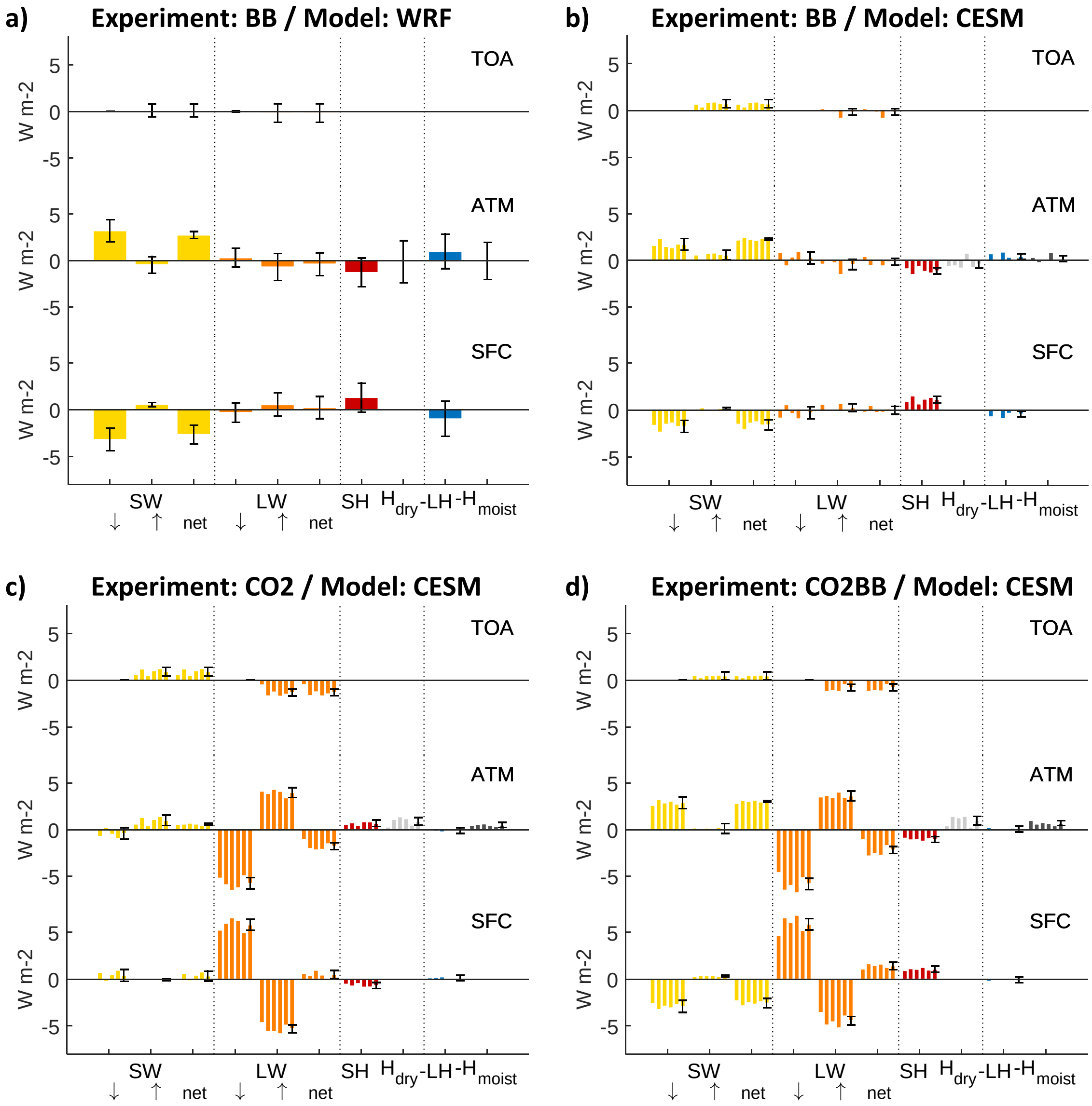

Supplementary Figure 9. Regional atmospheric energy budget for southern Africa. Atmospheric energy budget over the June to September season and averaged over the subregion (shown by the dashed rectangle in Fig. 1) for different models, ensemble members and experiments. Results are shown as differences from the BASE simulation, where CESM denotes the global climate model and WRF the regional model. At top-of-atmosphere (TOA) and surface (SFC), net downward energy fluxes have positive values. The energy budget of the atmosphere (ATM) is defined as the difference between the energy balance at TOA and SFC, except for the horizontal transport terms (H_{dry} and H_{moist}) which are positive when net energy transport is into the atmospheric column from the outside. For each of the terms in the CESM experiments, six bars are shown; one bar for each of the five ensemble members, and one bar which includes a black error bar, with the mean of the ensemble members. Error bars show the 95% confidence interval derived from a two-tailed Student's *t*-test. Abbreviations: SW, shortwave radiation; LW, longwave radiation; SH, sensible heat flux; LH, latent heat flux; H_{dry}, horizontal dry static energy flux; H_{moist}, horizontal moist static energy flux.

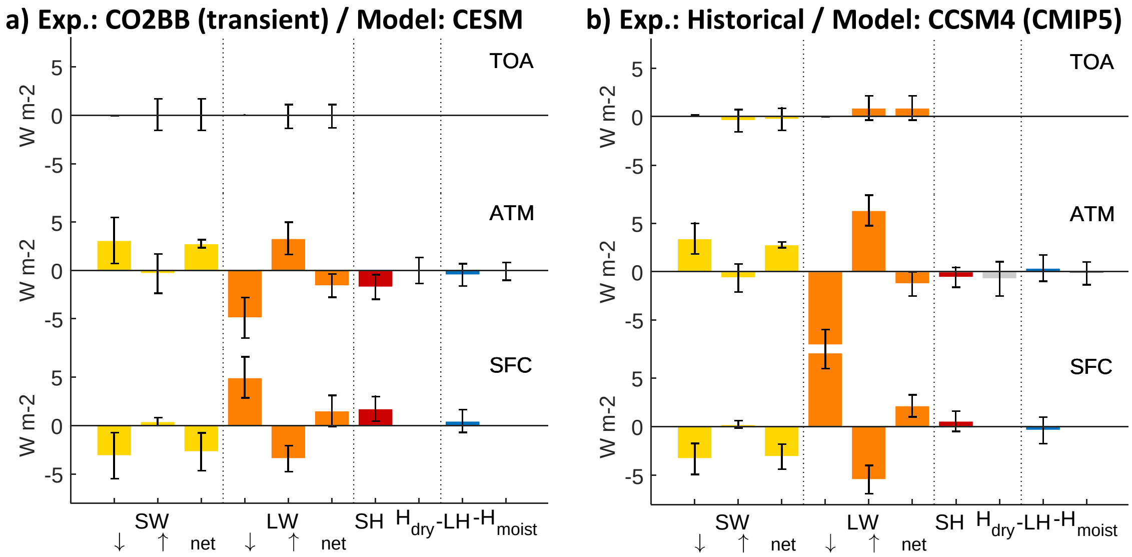

Supplementary Figure 10. Regional atmospheric energy budget for southern Africa. Same as Supplementary Figure 9, but for a CESM1.0.4 transient simulation which includes changes in CO_2 and BC/OC from biomass burning **(a)**, and from CCSM4 in the CMIP5 archive[3] of the "historical" simulation which includes all anthropogenic changes **(b)**. Results are shown as differences 1970:1999 – 1850:1879 in CESM1.0.4, and 1976:2005 – 1850:1879 in CCSM4 (CMIP5).

Supplementary Table 1. Correlation coefficient giving the spatial correlation of precipitation, and absolute change in precipitation, between various model experiments and observations in the subregion (shown by dashed rectangle in Fig. 1). Correlation coefficients are calculated for different horizontal resolutions and the grid dimensions in each of these resolutions are given in parenthesis. The top two rows show the correlation against the mean of GPCC observation data in the period 1981:2010, while the next four rows show the correlation against the difference in GPCC observations between the period 1981:2010 and 1901:1930, and the bottom four rows show the correlation against the difference in GPCC observations between the period 1981:2010 and 1951:1980. Bold values indicate that the correlation coefficients are significant ($p < 0.05$) according to a two-tailed Student's *t*-test.

			Correlation coefficients for different horizontal resolutions (and grid dimensions)			
Observations	Model	Experiment	0.5° (44x44)	2.5° (8x8)	5° (4x4)	10° (2x2)
GPCC mean 1981:2010	WRF	BASE	**0.92**	**0.97**	**0.99**	**0.99**
	CESM	BASE	**0.71**	**0.78**	**0.81**	0.93
GPCC Δ(1981:2010)-(1901:1930)	WRF	BB	**0.34**	**0.53**	**0.76**	0.82
	CESM	BB	**0.47**	**0.56**	**0.71**	0.87
	CESM	CO2	-0.027	-0.012	0.065	0.46
	CESM	CO2BB	**0.42**	**0.51**	**0.64**	0.83
GPCC Δ(1981:2010)-(1951:1980)	WRF	BB	**0.22**	**0.36**	**0.60**	0.86
	CESM	BB	**0.32**	**0.48**	**0.80**	**0.96**
	CESM	CO2	**0.19**	0.17	0.33	0.58
	CESM	CO2BB	**0.33**	**0.43**	**0.72**	0.87

References

1 Lamarque, J. F. *et al.* Historical (1850–2000) gridded anthropogenic and biomass burning emissions of reactive gases and aerosols: methodology and application. *Atmos. Chem. Phys.* **10**, 7017-7039, doi:10.5194/acp-10-7017-2010 (2010).

2 Skeie, R. B. *et al.* Anthropogenic radiative forcing time series from pre-industrial times until 2010. *Atmos. Chem. Phys.* **11**, 11827-11857, doi:10.5194/acp-11-11827-2011 (2011).

3 Taylor, K. E., Stouffer, R. J. & Meehl, G. A. An Overview of CMIP5 and the Experiment Design. *Bull. Amer. Meteorol. Soc.* **93**, 485-498, doi:10.1175/BAMS-D-11-00094.1 (2011).
